# Sexual Dysfunction in Ostomized Patients: A Systematized Review

**DOI:** 10.3390/healthcare9050520

**Published:** 2021-04-29

**Authors:** Mª Teresa García-Rodríguez, Adriana Barreiro-Trillo, Rocío Seijo-Bestilleiro, Cristina González-Martin

**Affiliations:** 1 Research in Nursing and Health Care, Institute of Biomedical Research of A Coruña (INIBIC), Complexo Hospitalario Universitario de A Coruña (CHUAC), SERGAS, As Xubias 84, 15006 A Coruña Universidade da Coruña (UDC), 15006 A Coruña, Spain; rocio.seijo.bestilleiro@sergas.es (R.S.-B.); cristina.gmartin@udc.es (C.G.-M.); 2 Complexo Hospitalario Universitario de A Coruña (CHUAC), SERGAS, Universidade da Coruña, 15006 A Coruña, Spain; abarreirotrillo@gmail.com

**Keywords:** digestive stoma, sexuality, sexual dysfunction, sexual behavior, review

## Abstract

The impact of an ostomy has a negative influence on sexuality. Healthcare professionals focus the care on surgery, and consider the sexual life is little relevant to the patient recovery. The aim of this systematized review is to give visibility to the sexual problems that ostomy patients have, to know what kind of sexual dysfunction occurs in this patients, to give information to the nursing staff about sexual disturbances and to recommend some resources to restart sexual activity. The research was conducted following de PRISMA guidelines and performed in several databases. Twelve papers were used to perform the systematized review. After ostomy, sexual dysfunction is different in men and women. It is related by the psychological aspects (low self-esteem, body image deterioration, etc.), the physical aspects (type of resection, complications, etc.) and the acceptance by the partner. A personalized sexual education focused on sexual problems that appear in ostomy patients is necessary to implement. In this way, adequate support, information and resources before and after surgery could be given for both, patients and their partners.

## 1. Introduction

The ostomy is an artificial communication between two organs or between a hollow viscus towards the exterior and this artificial opening is called stoma; it allows the elimination of waste products from the body. Depending on the level where the stoma is performed in the digestive system, we can speak of duodenostomy, jejunostomy, ileostomy or colostomy [1]. In Spain, there are more than 700,000 people with an ostomy and around 16,000 new cases are registered each year, with colostomies being performed most frequently (55.1%) followed by ileostomies (35.2%) [2].

The main cause to carry out a digestive ostomy is colorectal cancer, followed by inflammatory diseases, injuries, congenital diseases, malformations and intestinal obstructions [3]. This intervention affects self-image, bodily integrity, self-esteem and the ability to be socially related; thus, this type of procedure influences the sexuality of each individual who has one. Even so, despite the fact that 70% of patients’ report having an unsatisfactory sex life, the care and work in this field is mainly focused on surgical intervention (recovery, self-care, previous pathology), with only a low number of studies addressing an ostomy recipient’s sexuality [4]. Accordingly, a descriptive study was carried out, structured in the form of a systematized review and, for this, different databases were referred (PubMed, Scielo, Scopus and Dialnet).

## 2. Materials and Methods

The aim of this systematized review is to give visibility to the sexual problems that ostomy patients have, to know what kind of sexual dysfunction occurs in this patients, to give information to the nursing staff about sexual disturbances and to recommend some resources to restart sexual activity. This review was written following the Preferred Reporting Items for Systematic Reviews and Meta Analyses (PRISMA) protocol for systematic reviews [5].The research question chosen to carry out the systematized review was: How does ostomy affect the sex life of patients and their partners?

Our search focused on adult patients who have a digestive stoma (ileostomy or colostomy) and their sexual dysfunction. The outcomes were categorized as “sexual dysfunction in ostomized patients” and “sexual opinion of the patients’ spouses”. We set a timeframe of research published since 2008 to 2020. The retrieved articles were included if they were: (a) research reports conducted in adults, (b) written in English, Spanish or Portuguese and (c) published between 2008 and 2020. The exclusion criteria were: studies that deal with the digestive ostomized patients but do not address the sexuality aspect, unofficial documents, population information leaflets, letters to the editor, thesis, reviews or clinical practice guidelines.

Three nurse researchers independently identified the MeSh terms and used the keywords to develop a rigorous search strategy for this systematized review in different databases (PubMed, Scielo, Scopus and Dialnet) (Table 1). The literature search was started in May 2019 and ended in July 2019.

Three researchers independently screened the titles and abstracts of the studies found to identify those that met the inclusion criteria. Then, the articles that were not discarded were read in full text and assessed for their election. Disagreement over eligibility of studies was solved through discussion and by a fourth reviewer. To assess the quality of the articles, the scientific level of evidence designed by US Agency for Healthcare Research and Quality was used due to its simplicity and clarity [6]. There are five levels of scientific evidence depending on the type of study, establishing that the levels with a highest degree of scientific evidence are those that are in the highest part of the scale (such as meta-analyses and systematic reviews) and the lower levels are those that have less evidence and therefore less reliability. A data extraction sheet was developed and the data were extracted by the reviewers. Differences were discussed face to face, and when there was consensus the data were included. The variables that were taken into account were: author, country, year of publication, level of evidence, research design, simple size, type of ostomy, cause of stoma, measures and aim of the study. Data were synthesized and analyzed by the review authors, and the discrepancies were solved by consensus. The results were written as a descriptive narrative synthesis and tables were made to collect the variables taken into account.

## 3. Results

### 3.1. Selection and Characteristics of Sources of Evidence

**Figure 1:**
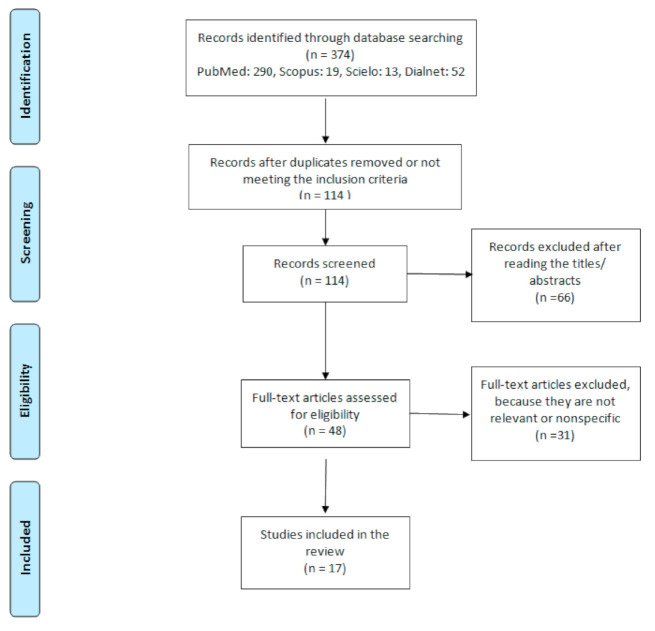
PRISMA flow diagram of search strategy.

After the bibliographic search, 374 articles were found. Two hundred and sixty were removed for being duplicates or not meeting the inclusion criteria. The remaining 114 articles were assessed by the authors in a primary review based on the reading of the titles and abstracts, discarding those articles (n = 66) that did not correspond to the subject of the review. Forty eight articles were read full text by authors and 31 were excluded because they did not focus on the objective of the work, were nonspecific or minimally relevant. A total of 17 articles were included in the systematic review for this study (Figure 1) published between 2008 and 2020.

Of the studies, five were qualitative; and the rest of them quantitative (descriptive, cases and controls, prospective, cross-sectional or randomized studies). According to the US Agency for Healthcare Research and Quality, the level of evidence of the most of the articles was IIb [7,8,9,10,11,12,13], followed by level III [14,15,16,17]. The rest of them were level IIa [18,19,20] and Ib [21,22,23]. The countries of the articles were: Turkey (n = 5) [7,12,13,15,21], Brazil (n = 4) [8,10,18,20], Netherlands (n = 3) [14,17,23], United States (n = 3) [9,11,22], Chile (n = 1) [19] and Ireland (n = 1) [16]. The type of stoma was mostly the colostomy, being the main cause for its realization the colorectal cancer. Several studies used a combination of instruments and different tools were used to assess sexuality. In six articles, the authors developed questionnaires for the study or used semi-structured interviews [7,8,10,11,12,18], and to assess the sexual dysfunction, the International Index of Erectile Function (IIEF) in men and the Female Sexual Function Index (FSFI) in women were the most used [9,13,15,22]. One author used the Golombok-Rust Inventory of Sexual Satisfaction (GRISS) to assess sexual dysfunction [21]. The anxiety, self-esteem, the quality of life or depression were also evaluated by some authors [9,13,14,15,16,17,19,20,22,23]. Only 2 articles studied the sexual (dys) function from the point of view of the spouse [7,18] (Table 2). Two articles show strategies to improve patients’ sex lives [21,22].

### 3.2. Results of Individual Sources of Evidence

The main outcomes were categorized in two parts:

- Sexual dysfunction in ostomized patients
- Sexual opinion of the patients’ spouses

Regarding *sexual dysfunction in ostomized patients*, fifteen articles have been found and two of them show strategies for improving patients’ sex lives [21,22]. The sexual affectation was different according to the sex of the patients. In men, the most common sexual dysfunction were erectile dysfunction and ejaculatory disorders [10,12,13,14,15,16,23], while in women were the dyspareunia and vaginal dryness [11,12,14,15,23]. Differences are also found according to the type of surgery [19] and treatment received [23]. In this sense, ostomy patients report a greater impact on their sex life than those who have had their anal sphincter preserved, who report a greater impact on their quality of life due to bowel dysfunction (diarrhea, incontinence) [19]. In terms of treatment, Wiltink’s study found that patients who underwent radiotherapy reported more erectile difficulties, more pain during intercourse and less sexual enjoyment [23]. Further, these alterations were found more frequently in patients with permanent colostomy and/or rectal cancer [14,15]. The impact of ostomy on desire and sexual activity was reflected in the fact that, in many cases, patients do not return to sexual intercourse [8,15,16,17], the presence of the ostomy interferes with intimacy [16] and sexual activity are seriously affect or damage by the lack of libido. Some authors relate the lack of desire and sexual satisfaction [9,10,13,15] with the change of body image, the rejection of the partner or the shame of having the ostomy [8,10,12,19,20]. All of these show a lack of self-esteem on the part of the patient, who will hinder his/her, relationships with others. Among the most common fears when having a sexual intercourse were the leakage from pouching system, the partner rejection and having the ostomy bag [8,10,11,12].

In relation to strategies to improve patients’ sex lives, Taylan et al. conducted a telephone follow-up of patients with bowel stoma and observed that when telephone counseling was offered for sexuality-related concerns, it significantly improved their sex lives [21]. Meanwhile, DuHamel et al. did an intervention with women with rectal and anal cancer that consisted of sessions on: sexual health, strategies to improve sexual life, methods of communication with the partner, and ended with support material (booklets, relevant referrals), observing that the group of women who participated in these sessions had less psychological stress and improved their sexual life [22].

*Sexual opinion of the patients’ spouses*. Only two articles were found in which the patients’ spouses give their opinion about the sexual activity [7,18]. In all of them, an alteration on the sexual performance was observed. The spouses report that interest in sex was less or was lost after ostomy and therefore, the intercourse frequency decreased or disappeared [7,18]. The main cause of this lack of sexual interest was the dissatisfaction of seeing the ostomy on their partner, which led to the decrease in sexual relations among the male spouses of ostomized women. [7]. This dissatisfaction was described not only at the sexual level, but also in social life because after the ostomy they participated less in social activities, travelled less and socialized less with their families. About 22.2% of the spouses considered ostomized patients’ sexual performance as unsatisfactory [18], and some of them, also complained about the lack of information on stoma management, as well as the psychological and social disorders and the implications of the stoma on daily life.

## 4. Discussion

Sexuality in ostomized patients is a little-studied subject. This is because ostomies are typically approached from a surgical perspective, with little consideration for the psychological and psycho-emotional impact it produces [3]. Through this review, it can be said that the sexual disorders’ causes are determined by two aspects: the psychological and the physical. On a psychological level, the stoma creation implies deterioration in body image. Furthermore, the lack of control over the body, the handling of the bag, the concern about the losses or the acceptance by the couple is added. In this way, it can be complicated to restart the sexual activity, as the self-esteem and the self confidence is dropping [3,8,10,12,19]. The main physical cause which affects sexual performance is the type of surgery, the treatment and/or the type of stoma [19,23]. Patients with permanent ostomies, rectal cancer, radiotherapy treatment or low resections, have more risk to suffer sexual dysfunction due to injuries in the pelvic nerves [13,15,19,23]. Both causes (physical and psychological) are supported by other studies. Albaugh et al. [24] also include in the physical causes, the cancer treatments, the tumor stage or the patient age [17,19]. Ribes Meliá [25] regard that both the psychological aspect and the type of resection will be decisive for sexual alteration. While Bonill et al. [26] confirm the importance of family support to restart the sexual live and improve the quality of life [20].

Before determining what resources are available to resume sexual activity, what’s really matter is the recognition by patients that they have problems in their sexual relationship. A situation assessment needs to be done [24]. For this, the healthcare staff must address the issue and, to promote the dialogue, different models have been developed, such as the PLISSIT model or the 5 A model [3,27].

After recognizing the existence of impaired sexual function, the next step is to assess it. There are different types of validated questionnaires and interviews, and there is no questionnaire specifically designed to assess sexual function in these patients, so questionnaires previously validated for other populations are used. There are general questionnaires such as the Derogatis Interview for Sexual Functioning (DISF/DISF-SR) [28] that measure the level and quality of sexual function or the Golombok-Rust Inventory of Sexual Satisfaction (GRISS) [21] which assesses the presence and severity of sexual problems. Even so, the authors consider that it is better to use specific questionnaires according to sex since, as has been said throughout the review, sexual dysfunction is not the same in men and women. Upon review, the most commonly used questionnaire in women was the Female Sexual Function Index (FSFI). Other questionnaires that could be used for ostomates women include, for instance, the Expanded Sexual Arousability Inventory (SAI-E) [29] or the Brief Index of Sexual Functioning (BISF-W) [30]. In men, the most commonly used questionnaire was the International Index of Erectile Function (IIEF). Other questionnaires that could also be used include, for example, the Brief Sexual Function Questionnaire (BSFQ) [31] or the Brief Sexual Function Inventory (BSFI) [32]. It would be difficult to determine the best method to assess sexual function. We believe that it would be useful to create a specific questionnaire for this population because sexual dysfunction is multifactorial and differs according to sex, type of resection, etc. In addition, a protocol should be standardized in which the ostomy nurse performs an initial assessment of sexual function before the operation, so the changes that occur afterwards can be seen. In this way, it may be possible to implement appropriate strategies for each couple and to determine whether the help of other professionals is needed.

Regarding the resources available, the recommendations given by Ribes Meliá [25] are to increase the security and control about the stoma, and their recommendations are: empty the pouch before sexual intercourse, use opaque pouch or stoma shutters, etc. Authors like Albaugh et al. [24] make recommendations for specific problems such as the decreased libido (cognitive behavioral treatment, sensate focus, etc.), vaginal dryness and pain (local estrogen therapy, pelvic floor physical therapy, etc.) or arousal disorders (vacum devices, implants, etc.). In addition to these recommendations, González Gómez [33] also addresses the issue of position during intercourse. In this sense, pressure on the abdomen or the colostomy bag should be avoided, cushions or pillows can be used to help find a more comfortable and pleasant posture or to use positions where the ostomy is a little more hidden.

After this review, the authors found many articles on ostomies, but very few studies on sexuality and sexual dysfunction in ostomy patients. In our opinion, more studies on sexual problems in the ostomy patient and more training for ostomy nurses about sexuality and sexual problems of patients are needed. In this way, quality care can be provided for the recovery of sexual life. Furthermore, despite the importance of the subject, there is no common protocol for its approach. A general guideline would be necessary to be created to establish, for instance, when is the best time to carry out educational and informative sexual intervention, the correct way to assess the sexual dysfunction in ostomized patients or how the healthcare personnel should ask questions about sexual problems. This way, the negative effects on sexual relations would be less [18].

We believe that sexual health education should be initiated prior to the creation of the stoma, thus encouraging the patient to address the issue after the ostomy has been performed.

### Limitations of This Study

The authors have made an effort to ensure rigor in conducting the systematized review. To this end, the PRISMA methodology has been followed and MeSh terms have been used for the bibliographic search. However, there are some limitations, such as the small number of studies dealing with the subject. Although at first the initial search returns a greater number of results, the established criteria have eliminated part of them. Excluding studies that are not in English, Spanish and Portuguese also represents a limitation since it does not allow us to know the existence of other articles in different languages that may contain relevant information on the subject to be discussed. In spite of these limitations, we consider that the subject is of great importance for the comprehensive recovery in these patients.

## 5. Conclusions

This document provides a new approach in the recovery of the stoma patient. This recovery should not only be seen from a surgical point of view but also from a perspective that include their sexual life. Finding excellence and achieving the humanization of care to report the well-being and satisfaction to these patients should be the main objective of nursing. Therefore, nursing management or leaders would be able to implement a personalized sexual education focused on sexual problems that appear in ostomy patients. In this way, adequate support, information and resources before and after surgery could be established for both, patients and their partners.

### Implications for Nursing Practice

This review aims to give visibility to the sexual problems that ostomy patients have. In most cases, patients, out of shame, do not ask for advice or help from professionals and this should be taken into account by the nurse managers because they have more possibilities to implement strategies that allow them to allocate resources, education and protocols to the practice. Therefore, protocols that can be used by nurses’ staff to provide coping strategies and resources to increase patient safety when engaging in sexual relations should be developed.

## Figures and Tables

**Table 1:** Keywords and Limits used to the Search Strategy.

Keywords	Limits
Enterostomy	Humans
Colostomy	Adults ≥ 18 years
Ileostomy	English
Stoma sexuality	Spanish
Sexual behavior	Portuguese
Sexual dysfunction	Free access to full text
Sex	
Body image	
Care	
Nursing	
Patient	
Behavior	

**Table 2:** Studies characteristics included in the review.

Author (Year) Country	Level of Evidence	Research Design	Simple Size	Type of Ostomy	Cause of Stoma	Measures	Aim
Cakmak (2010) Turkey [7]	IIb	Descriptive study	56	Colostomy	Rectal cancer	Questionnaire developed for the study	Assess the partners’ quality of life of patients undergoing sphincter sacrificing surgery for rectal cancer
Calcagno (2012) Brazil [8]	IIb	Qualitative study	10	NS	NS	Semi structured interviews	To know how the stoma interferes in the sexuality of ostomized women
Den Oudsten (2012) Netherland [14]	III	A population based study of cases and controls	1359	Colostomy	Colon cancer Rectal cancer	QLQ/CR38 FAS HADS	Compare colorectal cancer survivors with a normative population regarding sexual dysfunction
Milbury (2013) United States [9]	IIb	Descriptive, quantitative study	261	Colostomy	Colon cancer Rectal cancer	IIEF FSFI QLQ/CR38 Psychosocial questionnaires	Know the demographic, medical and psychosocial factors’ implication in sexual dysfunction due to colorectal cancer
Ozturk (2015) Turkey [15]	III	Retrospective case control study	84	Colostomy	NS	RSES IIEF, FSFI	Assess the sexual problems in patients with colostomies and its relationship with the self esteem
Alves (2013) Brazil [10]	IIb	Qualitative exploratory and descriptive research	11	NS	NS	Semi structured interviews	Describe the ostomized patients’ perception about their sexuality
Ramírez ^11^(2009) United States	IIb	Phenomenological study	30	Colostomy	Colorectal cancer	Semi structured open-ended interview	Examine the experiences of colorectal cancer females on the sexual challenges and their adaptation
Silva (2014) Brazil [18]	IIa	Prospective quantitative comparative case control study	108	Colostomy	Rectal cancer	Questionnaires developed for the study	How the partners of ostomized patients perceive the sexuality
Vural (2016) Turkey [12]	IIb	Phenomenological, qualitative design	14	Colostomy Ileostomy	Colorectal cancer Inflammatory bowel disease	Unstructured interview form prepared for the study	Describe the lived experiences of ostomized persons related to sexual function
Yilmaz (2017) Turkey [13]	IIb	Descriptive cross-sectional study	57	NS	Colon cancer Intestinal perforation	IIEF, IFSF SQOLS	Assess the effect of a stoma on sexual function and quality of life
Moreno (2019) Chile [19]	IIa	Cross-sectional cohort study	39	Colostomy	Rectal cancer	EQ-5D2 and add the dimension of sexual activity with three items	Compare the quality of life of patients undergoing abdominoperineal resection with patients undergoing sphincter preserving techniques
Davidson (2016) Irish [16]	III	Descriptive study	256	Colostomy Ileostomy Urostomy	Cancer Inflamatory bowel disease	Modified City of Hope Quality of life ostomy questionnaire	Assess the quality of life, wellbeing and care needs of irish ostomates
Taylan (2019) Turkey [21]	Ib	Randomized controlled quasi experimental study	70	Colostomy Ileostomy	Cancer Inflamatory bowel disease	GRISS	Determine the effect of telephone counseling on the sexual lives of individuals with bowel stoma
DuHamel (2016) USA [22]	Ib	Randomized controlled trial	70	Colostomy	Rectal and anal cancer	FSFI, IES-R, BSI EORTC-QLQ-C30	Assess the efficacy of a telephone-based four session Cancer Survivorship Intervention-Sexual Health
Wiltink (2014) Netherlands [23]	Ib	Multicenter randomized trial	478	Colostomy	Rectal cancer	EORTC-QLQ-C30, C29, CR38, CX24, PR25	Assess the health-related quality of patients treated with total mesorectal excision
Alegre Salles (2014) Brazil [20]	IIa	Cross-sectional epidemiological study	30	Colostomy Ileostomy	Colorectal cancer Inflammatory bowel disease Traumatic bowel perforation	Interviews WHOQOL-BREF	Evaluate the quality of life in ostomized patients
Orsini (2012) Netherlands [17]	III	Descriptive study	143	Colostomy	Rectal cancer	EORTC QLQ-C38 SF-36, SCQ	Investigate the impact of a stoma on the quality of life of older rectal cancer patients

**NS**: Not Specified; **BAS**: Burden Assessment Scale; **BDI**: Beck Depression Inventory; **STAI**: State-Trait Anxiety Inventory; **FAS**: Fatigue Assessment Scale; **HADS**: Hospital Anxiety and Depression Scale; **FSFI**: Female Sexual Function Index; **IIEF**: International Index of Erectile Function; **SQOLS**: Stoma Quality of Life Scale; **QLQ-CR38**: Colorectal Cancer Specific Quality of Life Questionnaire; **RSES**: Rosenberg Self Esteem; **EQ-5D2**:EuroQol-5 Dimensional; **GRISS**: Golombok-Rust Inventory of Sexual Satisfaction; **IES-R**: Impact of Events Scale-Revised; **BSI**: Brief Symptom Inventory; **EORTC-QLQ**: European Organization for Research and Treatment of Cancer Core Quality of Life Questionnaire; **WHOQOL-BREF**: World Health Organization Quality of Life Short Form; **SCQ**: Self-administered Comorbidity Questionnaire.

## Data Availability

Data sharing not applicable.
